# Retrospective Analysis of Radiological Recurrence Patterns in Glioblastoma, Their Prognostic Value And Association to Postoperative Infarct Volume

**DOI:** 10.1038/s41598-018-22697-9

**Published:** 2018-03-14

**Authors:** Stefanie Bette, Melanie Barz, Thomas Huber, Christoph Straube, Friederike Schmidt-Graf, Stephanie E. Combs, Claire Delbridge, Julia Gerhardt, Claus Zimmer, Bernhard Meyer, Jan S. Kirschke, Tobias Boeckh-Behrens, Benedikt Wiestler, Jens Gempt

**Affiliations:** 1 Department of Neuroradiology, Klinikum rechts der Isar, Technische Universität München, Munich, Germany; 2 Department of Neurosurgery, Klinikum rechts der Isar, Technische Universität München, Munich, Germany; 3 Department of Radiology, University Hospital, LMU, Munich, Germany; 4 Department of Radiation Oncology, Klinikum rechts der Isar, Technische Universität München, Munich, Germany; 5 Department of Neurology, Klinikum rechts der Isar, Technische Universität München, Munich, Germany; 6 Department of Neuropathology, Klinikum rechts der Isar, Technische Universität München, Munich, Germany; 7 Institute of Innovativ Radiotherapy (iRt), Department of Radiation Sciences (DRS) Helmholtz Zentrum München, Ingolstädter Landstraße Neuherberg, Munich, Germany; 8 Deutsches Konsortium für Transnationale Krebsforschung (DKTK), Partner Site Munich, Munich, Germany

## Abstract

Recent studies suggested that postoperative hypoxia might trigger invasive tumor growth, resulting in diffuse/multifocal recurrence patterns. Aim of this study was to analyze distinct recurrence patterns and their association to postoperative infarct volume and outcome. 526 consecutive glioblastoma patients were analyzed, of which 129 met our inclusion criteria: initial tumor diagnosis, surgery, postoperative diffusion-weighted imaging and tumor recurrence during follow-up. Distinct patterns of contrast-enhancement at initial diagnosis and at first tumor recurrence (multifocal growth/progression, contact to dura/ventricle, ependymal spread, local/distant recurrence) were recorded by two blinded neuroradiologists. The association of radiological patterns to survival and postoperative infarct volume was analyzed by uni-/multivariate survival analyses and binary logistic regression analysis. With increasing postoperative infarct volume, patients were significantly more likely to develop multifocal recurrence, recurrence with contact to ventricle and contact to dura. Patients with multifocal recurrence (Hazard Ratio (HR) 1.99, P = 0.010) had significantly shorter OS, patients with recurrent tumor with contact to ventricle (HR 1.85, P = 0.036), ependymal spread (HR 2.97, P = 0.004) and distant recurrence (HR 1.75, P = 0.019) significantly shorter post-progression survival in multivariate analyses including well-established prognostic factors like age, Karnofsky Performance Score (KPS), therapy, extent of resection and patterns of primary tumors. Postoperative infarct volume might initiate hypoxia-mediated aggressive tumor growth resulting in multifocal and diffuse recurrence patterns and impaired survival.

## Introduction

Glioblastoma (GB) is the most common malignant brain tumor of adults. Despite aggressive therapy including surgery and combined radiochemotherapy according to the Stupp protocol almost all patients show early recurrence and fatal outcome^1,2^. Several prognostic factors have been established, including age, Karnofsky Performance Score (KPS), extent of resection and molecular markers^3–8^. Studies showed that postoperative infarct volume is - beside its impact on postoperative functional independence - also an independent prognostic factor for overall survival^9–11^. A recent study demonstrated that patients with ischemia after glioblastoma surgery are more likely to develop diffuse and more distant tumor recurrence, suggesting that postoperative hypoxia might introduce an infiltrative tumor growth with distinct recurrence patterns^12^. In fact, most GB show local tumor recurrence, with only about 10% having distant tumor growth at first recurrence^13,14^. However, distant tumor recurrence and tumor recurrence close to the ventricle are known to be associated with impaired overall survival^15–17^.

The authors hypothesize (i) that there might be correlations between postoperative infarct volume and recurrence patterns and (ii) that these radiological recurrence patterns are associated with patients’ prognosis (overall survival (OS) and post-progression survival (PPS)).

## Patients and Methods

Ethical approval was obtained by the local ethics committee for this retrospective, non-interventional retrospective single-center study. Informed consent was waived by the local ethics committee due to the retrospective design of this non-interventional study. The study was conducted in accordance with the ethical standards of the 1964 Declaration of Helsinki and its later amendments^18^.

### Patient population

526 consecutive glioblastoma patients treated in our institution between January 2006 and March 2016 were retrospectively analyzed. 251 patients have been previously reported. This prior article dealt with the impact of postoperative infarct volume on overall survival^9^. From this previous cohort 129 patients met our inclusion criteria: surgery (biopsies were excluded) for a newly diagnosed glioblastoma (WHO IV) without previous treatment (eg surgery or radiochemotherapy for a lower grade glioma), early postoperative MRI (<72 h) including postoperative diffusion weighted imaging (DWI) and tumor recurrence (with available MRI) during the observation period. Exclusion criteria comprised previous treatment of the glioma/glioblastoma, missing postoperative diffusion weighted imaging or missing tumor recurrence.

Patients’ medical charts were analyzed for the following parameters by a neurosurgeon, blinded to radiological recurrence patterns: pre- and postoperative KPS, KPS at date of recurrent disease (postoperative KPS in case of second surgery), neurological status after initial surgery, date of surgery, 5-aminolevulinic acid (5-ALA)/intraoperative neuromonitoring (available in 128/129 patients), date of death or date of last contact in alive patients. Date of tumor progression was defined as date of MRI with progressive disease according to the RANO criteria^19^ and in an interdisciplinary consensus of neurosurgery, neuroradiology, radiation oncology, neuropathology, nuclear medicine and neurooncology. OS and progression free survival (PFS) were calculated from the date of surgery. PPS was calculated from date of tumor recurrence (MRI) to death/last contact. Treatment regimens after initial surgery/tumor diagnosis as well as after tumor recurrence were recorded for each patient.

Histopathological analysis was performed according to the WHO criteria of CNS tumors^20^.

Methylation status of O^6^-methylguanine DNA methyltransferase (MGMT) was analyzed quantitatively (percentage) and available in 95/129 cases. Proliferation index Ki67 was calculated by the ratio of labeled to unlabeled nuclei (percentage) (MIB-1 antibody, anti-Ki-67, 1:50, Dako, Hamburg, Germany) and available in 60/129 patients.

### Magnetic resonance imaging

Early postoperative MRI scans were performed in the Department of Neuroradiology at a 3 Tesla MRI scanner, either Philips Achieva or Philips Ingenia (Philips Medical Systems, The Netherlands B.V.) or Siemens Verio (Siemens Healthcare, Erlangen, Germany). Postoperative images included DWI or Diffusion Tensor Imaging (DTI), from which apparent diffusion coefficient (ADC) maps were calculated automatically (Achieva: DWI: 2:10 min, 7221/55 ms, 2 × 2 × 2 mm; DTI: 6.26 min, 10728/55 ms, 2 × 2 × 2 mm; Ingenia: DTI: 4.58 min, 16119/61 ms, 2 × 2.04 × 2 mm; Verio: DWI: 2:30 min, 5700/91 ms, 1.4 × 1.4 × 4 mm, DTI: 1:28 min, 3600/95 ms, 1.8 × 1.8 × 4 mm). Postoperative images as well as preoperative and follow-up images also included T2-weighted fluid attenuated inversion recovery (FLAIR) images (Achieva: 2D FLAIR: 3:00 min, TR/TE: 12000/140 ms, spatial resolution 0.45 × 0.45 × 4 mm; 3D FLAIR: 4:52 min, 4800/278 ms, 1.04 × 1.04 × 1.12 mm; Ingenia: 2D FLAIR: 3:00 min, 12000/140 ms, 0.9 × 0.95 × 4 mm, 3D FLAIR: 4:34 min; 4800/302 ms, 1.12 × 1.12 × 1.12 mm; Verio: 2D FLAIR: 3:44 min, 8560/136 ms, 0.8 × 0.4 × 4 mm, 3D FLAIR: 5:52 min, 5000/395 ms, 1 × 1 × 1 mm) and T1w with and without contrast agent (Achieva: 2D T1: 2:53 min, 530/10 ms, 0.45 × 0.45 × 4 mm; 3D T1: 5:55 min, 9/4 ms, 1 × 1 × 1 mm, Ingenia: 2D T1: 3:16 min, 590/10 ms, 0.9 × 1.12 × 4 mm; 3D T1: 5:59 min, 0.99 × 1.05 × 1 mm, Verio: 2D T1: 4:02 min, 2000/9 ms, 0.9 × 0.7 × 4 mm; 3D T1: 4:18 min, 1900/2.45 ms, 1.1 × 1.1 × 1 mm). Magnograf® was administered intravenously (0.2 ml/kg, 0.5-1 ml/sec), image acquisition started 90 seconds after administration of the contrast agent.

### Image analysis

Image analysis was performed by a neuroradiologist (SB, 6 years of experience) and a second neuroradiologist (BW, 6 years of experience) for 60 randomly selected patients to assess interrater-reliability, blinded to patients’ outcome. Pre- and postoperative tumor volume (including the volume of the contrast-enhancing tumor part) was measured by manual segmentation using IPlannet (IPlan 3.0 cranial planning software, Brainlab AG, Munich, Germany) in T1-weighted images after contrast agent. Extent of resection was calculated by pre- and postoperative tumor volume and defined as complete resection (100%), near-total resection (>90%) and subtotal resection (<90%).

Manual segmentation was performed by a neuroradiologist (SB). To assess interrater-reliability, a second rater (MB, 2 years of experience) performed manual segmentation of pre- and postoperative tumor volume of 29 patients (n = 58).

Postoperative ischemic lesions were also manually segmented by using IPlannet by a neuroradiologist (SB). Ischemia was defined as hyperintensity on b1000 images of DWI with a corresponding hypointensity on ADC maps as described before^9^. Postoperative changes including edema and methaemoglobin were excluded from these volumes by fusion of T2-weighted FLAIR- and gradient-echo sequences to DWI images.

**Figure 1:**
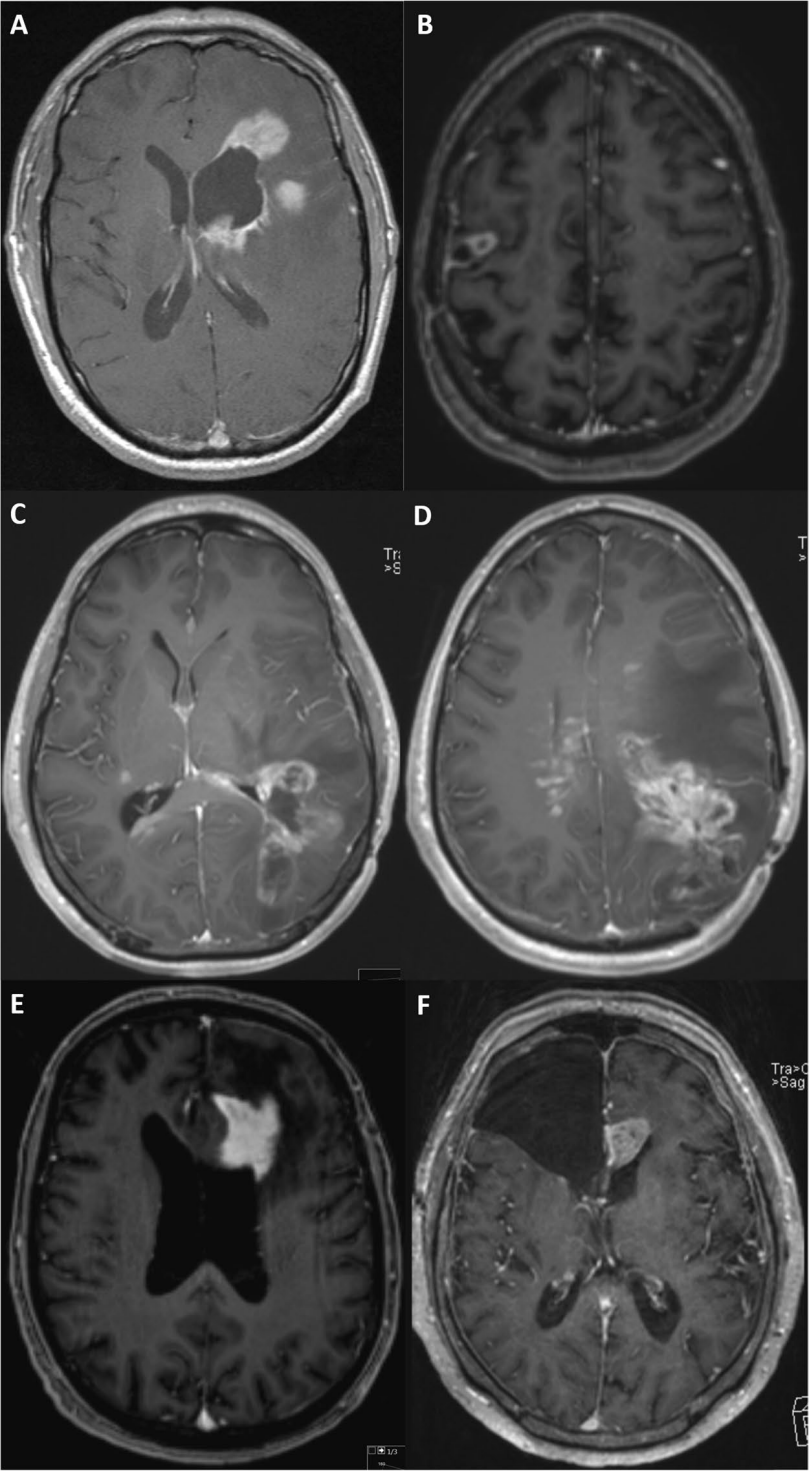
Examples of different radiologic recurrence patterns: (**A**) shows a multifocal, extensive tumor growth with contact to the ventricle, (**B**) a local, circular recurrence pattern, (**C** and **D**) an extensive multifocal, local and distant tumor growth with contact to ventricle and ependymal spread, (**E** and **F**) an extensive recurrence with contact to the ventricle.

For primary tumors and tumor recurrence the following parameters were obtained:, uni- or multifocal (≥2 enhancing lesions) recurrence, contact to dura, contact to the ventricle, ependymal tumor spread, haemorrhage. For contrast enhancement the following recurrence patterns were defined: invasive or displacing growth, homogenous or inhomogenous enhancement, with or without sharp demarcation, circular, garland-like, extensive, streaky or solid enhancement (Fig. 1). To simplify the analysis circular and garland-like contrast enhancement were summarized as well as extensive/streaky and solid enhancement. For tumor recurrence the location of recurrence was further recorded: local (with contact to the resection cavity) or distant (without contact) to the resection cavity.

The patterns of contrast enhancement were used for local and/or distant tumor recurrence.

### Statistical analysis

Statistical analysis, including descriptive data analysis, was performed using IBM SPSS Statistics Version 23.0 (SPSS Inc., IBM Corp., Armonk, NY, USA). Non-normally distributed data are shown as median and interquartile range (IR), normally distributed variables as mean and standard deviation. Correlations between patterns of primary and recurrent tumors were analyzed using the Fisher’s exact test. Differences between two groups were analyzed by the Mann-Whitney U test. To assess relations between postoperative infarct volume and recurrence patterns a binary logistic regression model was performed for recurrence pattern as dependent variable and infarct volume as covariate (cm³). OS distributions were compared using Kaplan-Meier estimates (Log-rank) and a Cox proportional hazard regression model for multivariate survival analysis. For calculation of interrater-reliability for qualitative analysis the cohen’s kappa (standard error = r^a^) was used, for quantitative analysis the intraclass correlation coefficient (ICC). Differences with a type one error probability of less than 0.05 were considered statistically significant.

## Results

### Patient population

129 consecutive patients (52 female, mean age at initial diagnosis 59.4 years+/−12.8) who underwent surgery for a newly diagnosed glioblastoma (WHO IV) were retrospectively included in this single-center study (Table 1). The median preoperative KPS was 80 (IR 70-90), the median postoperative KPS was 80 (IR 70-90), the KPS at date of recurrent disease (postoperative KPS in case of second surgery) was 70 (IR 60-80). 40/129 patients had temporary neurological deficits after surgery, 18 presented with permanent deficits. Histopathological analysis indicated MGMT promoter methylation in 36/95 cases. Median Ki67 was 20% (15–25). 51/128 patients were operated using 5-ALA, 102/128 patients using intraoperative neuromonitoring. 94/129 patients died during follow-up. Median overall survival was 16.1 months (95% CI 13.8–18.4) after initial diagnosis of a glioblastoma, median time to first tumor recurrence was 7.2 months (95% CI 5.6–8.8). Recurrent disease was proven histologically in 58/129 cases, by MR analysis according to the RANO criteria in 48/129 cases and by advanced imaging methods including O-(2-[18 F]-Fluoroethyl)-L-Tyrosine-Positron Emission Tomography (FET-PET) or perfusion weighted imaging in 23/129 cases.

**Table 1:** Baseline patient and tumor characteristics.

Age at date of initial diagnosis	59.4 y ( + /−12.8)
Sex, female	52/129 (40.3%)
KPS preoperative	80 (70–90)
KPS postoperative	80 (70–90)
KPS at recurrence	70 (60–80)
MGMT-methylation	36/95 (37.9%)
Ki67	20.0% (15.0–25.0)
Death during FU	94/129 (72.9%)
OS after ID	16.1 months (95% CI 13.8–18.4)
PFS	7.2 months (95% CI 5.6–8.8)
PPS	7.5 months (95% CI 6.4–8.6)
Preoperative tumor volume	29.6 cm³ (11.7–54.0)
Postoperative tumor volume	0.0 cm³ (0.0–0.9)
Postoperative infarct volume	2.0 cm³ (0.8–5.3)
Complete tumor resection	65/129 (50.4%)
Near-total tumor resection (>90%)	49/129 (38.0%)
Subtotal tumor resection (<90%)	15/129 (11.6%)

Normally distributed variables shown as mean + /− standard deviation, non-normally distributed as median (interquartile range);

OS, overall survival, ID: initial diagnosis, PFS: progression free survival, PPS: post-progression survival.

Almost all patients received adjuvant therapy after initial surgery (124/129), 106/129 patients received therapy for recurrent disease. After initial surgery, most patients (96/124) received combined radiochemotherapy according to the Stupp protocol with six weeks of combined radiochemotherapy followed by six months of chemotherapy with temozolomide^2^. At our department adjuvant therapy usually starts 2-4 weeks after surgery. Table 2 lists the therapy regimes after initial surgery and after diagnosis of recurrent disease.

**Table 2:** Adjuvant therapy.

**Initial adjuvant therapy**	
- Stupp scheme	96/129 (74.4%)
- only chemotherapy	8/129 (6.2%)
- only radiotherapy	24/129 (18.6%)
- bevacizumab	4/129 (3.1%)
- Chlorethyl-Cyclohexyl-Nitroso-Urea (CCNU)	3/129 (2.3%)
**Therapy after first recurrence**	
- surgery	56/129 (43.4%)
- chemotherapy	80/129 (62.0%)
- radiotherapy	47/129 (36.4%)
- bevacizumab	26/129 (20.2%)
- CCNU	13/129 (10.1%)

The intraclass-correlation coefficient for volumetric measurement showed an excellent agreement between the two raters (0.988 [95% CI 0.979–0.993], p < 0.0001). Median preoperative tumor volume was 29.6 cm³ (IR 11.7–54.0), median postoperative tumor volume 0.0 cm³ (IR 0.0-0.9). 65/129 patients had gross total tumor resection, 49/129 near-total tumor resection (>90%) and 15/129 subtotal tumor resection (<90%). No significant association was observed between the use of 5-aminolevulinic acid/intraoperative neuromonitoring and extent of resection/infarct volume. Postoperative ischemic changes were present in 113/129 patients including also rim-like infarctions surrounding the resection cavity. Median postoperative infarct volume was 2.0 cm³ (IR 0.8–5.3). Patients with insular tumors showed significantly larger infarct volumes (6.50 cm³ vs. 1.13 cm³; P = 0.040).

### Primary tumors and recurrence patterns

Most of the primary tumors showed typical garland-like contrast enhancement (112/129) with invasive growth (121/129) and without sharp demarcation (116/129). Contact to the dura was observed in 73/129 patients, contact to ventricle in 62/129 patients (Table 3). 8/129 primary tumors were located in the insula.

**Table 3:** Patterns of primary tumors and tumor recurrence.

**Primary tumor**	
Multifocal tumor	39/129 (30.2%)
Contact to ventricle	62/129 (48.1%)
Contact to dura	73/129 (56.6%)
Ependymal spread	0/129 (0%)
Hemorrhage	120/129 (93.0%)
**Pattern of contrast enhancement**	
- circular/garland-like	112/129 (86.8%)
- extensive/solid/streaky	17/129 (13.2%)
- homogenous	121/128 (94.5%)
- inhomogenous	7/128 (5.5%)
with sharp demarcation	13/129 (10.1%)
without sharp demarcation	116/129 (89.9%)
invasive	121/129 (93.8%)
displacing	8/129 (6.2%)
**Tumor recurrence**	
**Location of recurrence**	
- local	72/129 (55.8%)
- distant	13/129 (10.1%)
- local and distant	44/129 (34.1%)
Multifocal recurrence	85/129 (65.9%)
Contact to ventricle	77/129 (59.7%)
Contact to dura	64/129 (49.6%)
Ependymal spread	14/129 (10.9%)
Hemorrhage	5/129 (3.9%)
**Pattern of contrast enhancement (n = 128)**	
- circular/garland-like	59/128 (46.1%)
- extensive/solid/streaky	69/128 (53.9%)
- homogenous	30/128 (23.4%)
- inhomogenous	98/128 (76.6%)
with sharp demarcation	24/129 (18.6%)
without sharp demarcation	105/129 (81.4%)
invasive	124/129 (96.1%)
displacing	5/129 (3.9%)

Cohen’s kappa showed excellent agreement between the two readers for analysis of location of tumor recurrence (0.868, r^a^ = 0.063, P < 0.001), multifocal tumor recurrence (0.836, r^a^ = 0.079, P < 0.001), contact to dura (0.900, r^a^ = 0.056, P < 0.001), contact to ventricle (0.928, r^a^ = 0.050, P < 0.001), ependymal spread (1.000, r^a^ = 0.000, P < 0.001), hemorrhage (1.000, r^a^ = 0.000, P < 0.001), homogenous/inhomogenous enhancement (1.000, r^a^ = 0.000, P < 0.001) and pattern of contrast enhancement (0.896, r^a^ = 0.058, P < 0.001). Moderate agreement was shown for the parameters invasive tumor growth (0.700, r^a^ = 0.163, P < 0.001) and demarcation (0.632, r^a^ = 0.115, P < 0.001).

Local tumor recurrence (at the site of former surgery) was observed in 72/129 patients. While 44 patients had a combined local and distant recurrence, we observed a sole distant tumor recurrence in 13 patients. 85/129 patients presented with multifocal tumor recurrence, in 77/129 recurrent tumor mass had contact to the ventricles. 64/129 patients showed tumor recurrence with contact to the dura, an ependymal tumor spread was observed in 14/129 patients. Almost all patients (128/129) had a contrast-enhancing tumor recurrence. The following contrast-enhancing patterns were observed: inhomogeneous in 98/128 patients, infiltrative in 124/129 patients, without sharp demarcation in 105/129 patients and circular/garland-like in 59/128 patients. 5/129 patients presented with hemorrhage at recurrent disease. Table 3 lists the recurrence patterns of all patients.

Significant correlations between primary and recurrent tumors were observed for the following parameters: contact to dura (P = 0.021), contact to ventricle (P < 0.01) and pattern of contrast enhancement (P = 0.017). No significant correlations were observed for multifocal growth (P = 0.106) and ependymal spread (P = 1.0).

### Recurrence patterns and postoperative infarct volume

**Figure 2:**
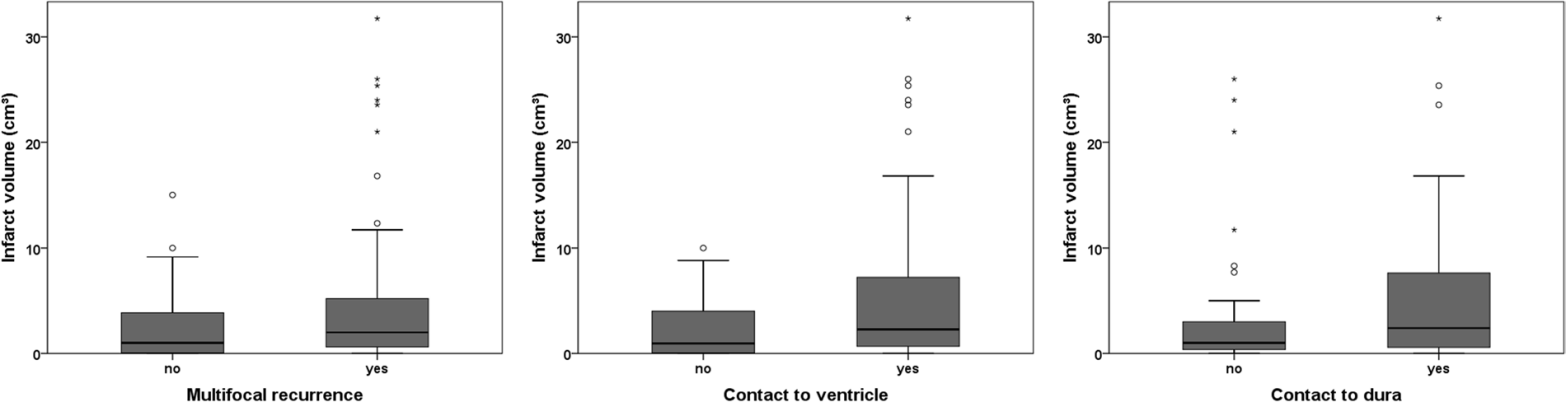
Boxplots for infarct volume and multifocal recurrence/contact to ventricle/contact to dura.

**Figure 3:**
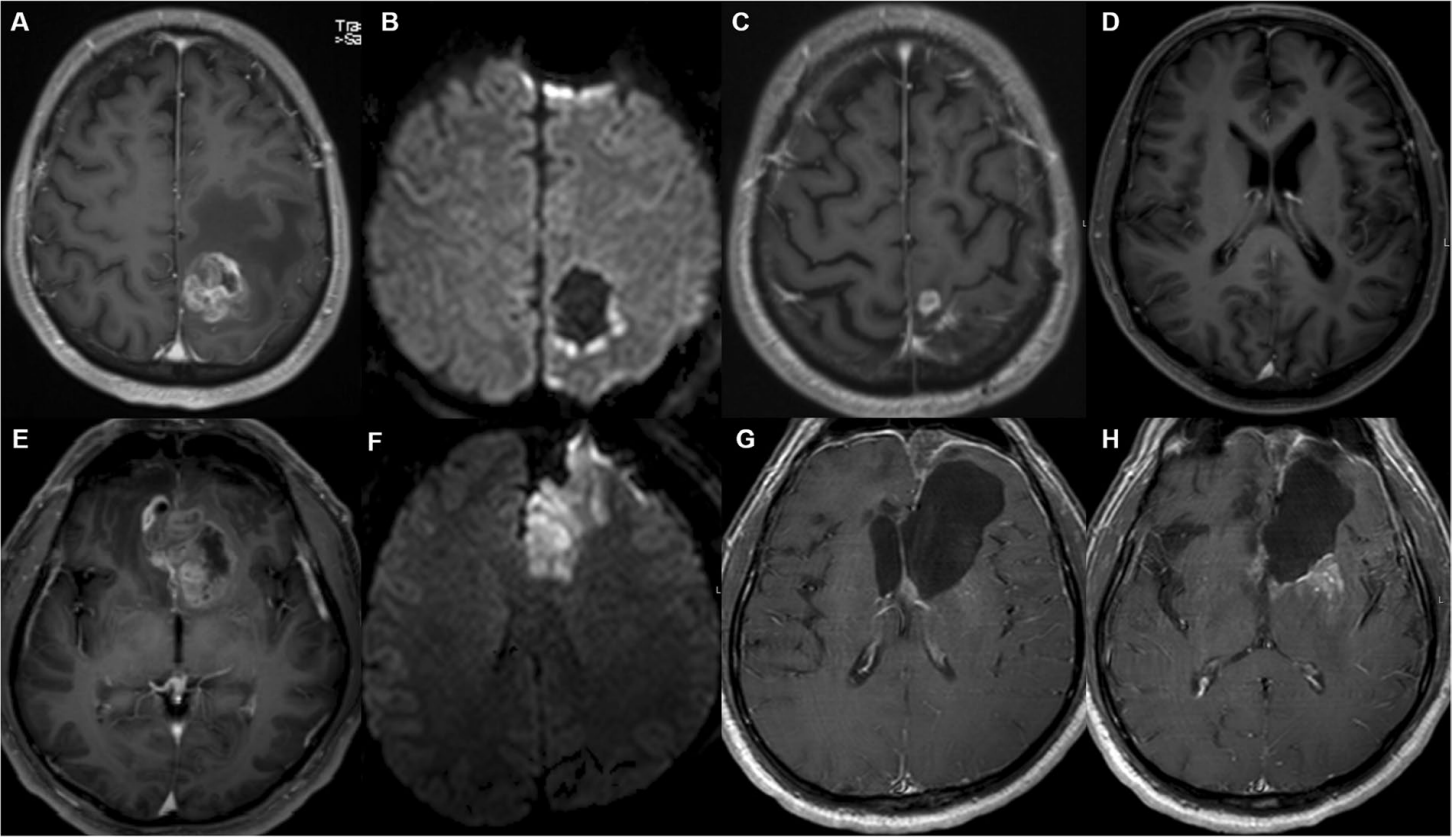
The first row (**A**–**D**) shows preoperative T1w contrast images (**A**), postoperative DWI (**B**) and T1w contrast images at date of tumor recurrence (**C**,**D**)). In the postoperative DWI only rim-like DWI-hyperintensity without territorial ischemia was present, in this case a local unifocal recurrence pattern (**C**), but not multifocal recurrence with contact to ventricle (**D**) was observed. The second row (**E**–**H**) shows pre- (**E**), postoperative (**F**) and follow-up (**G**,**H**) images of another patient. (**F**) shows a larger DWI-hyperintensity in the territory of the anterior cerebral artery. In this case a multifocal tumor recurrence with contact to the ventricle was observed.

A binary logistic regression analysis revealed that with increasing postoperative infarct volume, patients were significantly more likely to develop multifocal tumor recurrence (OR 1.10 [95% CI 1.00–1.21] per cm³ ischemia volume, P = 0.040) as well as contact to ventricle (OR 1.14 [1.03–1.26], P = 0.012) and contact to dura (OR 1.07 [1.01–1.15], P = 0.029) (Fig. 2). No significant association was observed for location of tumor recurrence (OR 1.03 [0.98–1.09], P = 0.186), ependymal spread (OR 1.01 [0.95–1.09], P = 0.726), pattern of contrast enhancement (OR 1.01 [0.96–1.06], P = 0.627), homogenous/inhomogeneous contrast enhancement (OR 1.05 [0.97–1.14], P = 0.210), invasive/displacing tumor growth (OR 1.04 [0.87–1.24], P = 0.675) and demarcation (OR 1.02 [0.95–1.09], P = 0.627). Figure 3 shows examples of two patients.

Extent of resection did not show a significant correlation to infarct volume (Spearman: r = 0.078, P = 0.381). Further, binary logistic regression analysis did not show associations between extent of resection and aggressive recurrence patterns.

### Overall survival

#### Univariate model

**Figure 4:**
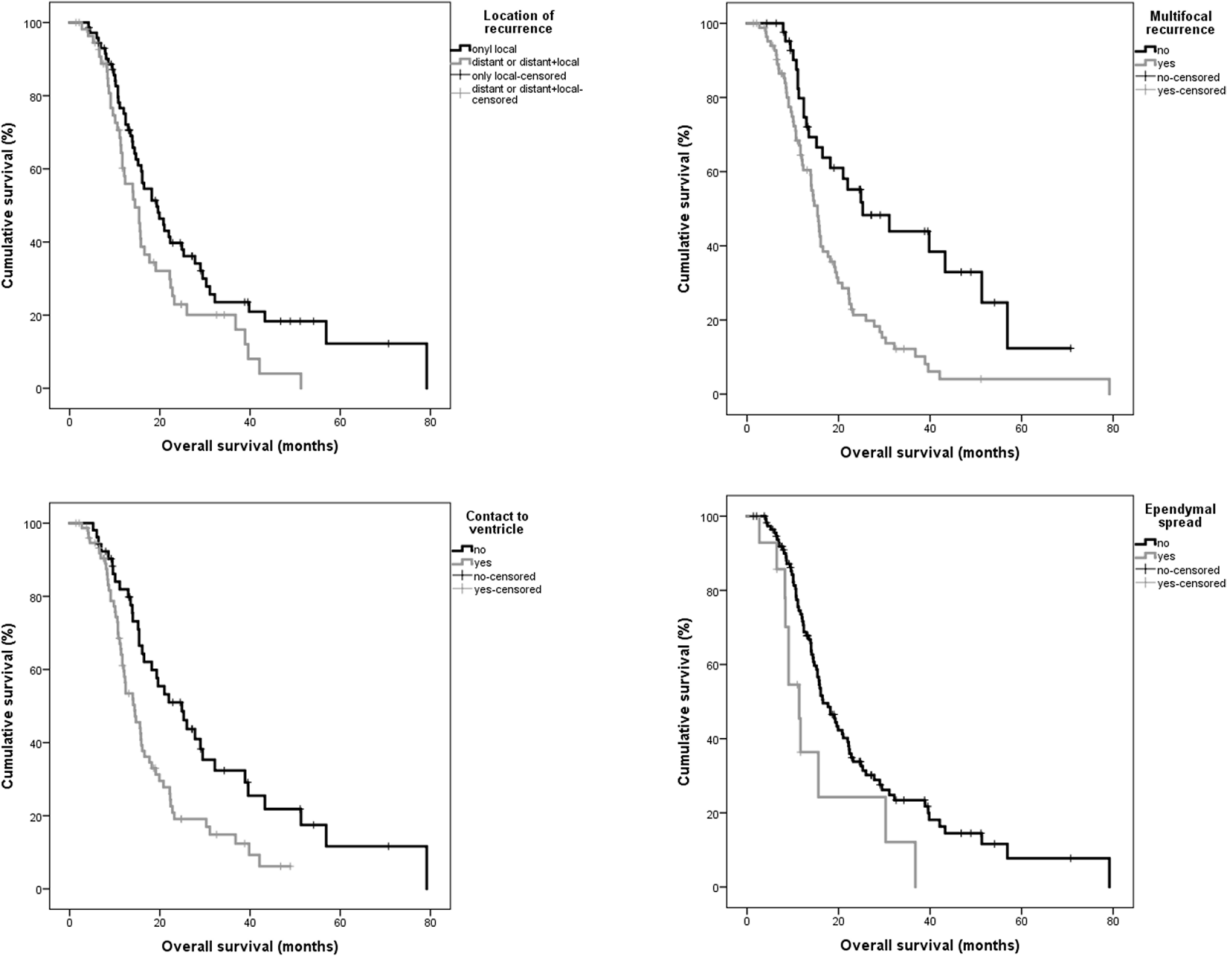
Overall survival estimates (Kaplan-Meier) for different recurrence patterns.

In univariate analysis the following parameters were significantly associated with survival: extent of resection (P < 0.001), postoperative KPS (<80/>/ = 80) (P = 0.027), KPS at tumor recurrence (P = 0.001), MGMT-methylation status (P = 0.013), adjuvant therapy after initial surgery (P = 0.003) and therapy for recurrent disease (P < 0.001). The following recurrence patterns were associated with impaired prognosis: multifocal tumor recurrence (P < 0.001), contact to ventricle (P = 0.002), ependymal spread (P = 0.019) and distant tumor recurrence (P = 0.012) (Fig. 4). Pattern of contrast enhancement (P = 0.132) and the presence of a new neurological deficit after surgery (temporary deficits: P = 0.733; permanent deficits P = 0.484) showed no significant association to patients’ prognosis. Multifocal growth and contact to ventricle for primary tumors missed statistical significance (P = 0.086/P = 0.059).

#### Multivariate model

The multivariate survival model included the following parameters: age at surgery, extent of resection, dichotomized KPS at tumor recurrence (<80/>/ = 80), therapy for tumor recurrence, multifocal growth and contact to ventricle of primary tumors, multifocal recurrence, contact to ventricle and ependymal spread at recurrence and location of tumor recurrence (distant/local). A significant impact on overall survival was present for extent of resection (<90% vs. >90%) (Hazard ratio (HR) 4.68 [95% confidence interval 2.42–9.02], P < 0.001), absence of further therapy for recurrent disease (HR 4.79 [2.46–9.35], P < 0.001) and multifocal tumor recurrence (HR 1.99 [1.18–3.34], P = 0.010) (Table 4).

**Table 4:** Multivariate survival analysis (Cox regression model)

Parameter	Hazard Ratio	95% CI	*P* value
***Overall survival***			
Age	1.01	0.99–1.03	0.411
Extent of resection (<90% vs. >90%)*	4.68	2.42–9.02	<0.001
No therapy for recurrent disease*	4.79	2.46–9.35	<0.001
KPS at recurrent disease (<80/>/=80)	1.29	0.77–2.14	0.334
Multifocal primary tumor	1.25	0.76–2.07	0.382
Primary tumor: contact to ventricle	0.98	0.57–1.70	0.948
Multifocal recurrence*	1.99	1.18–3.34	0.010
Recurrence: contact to ventricle	1.70	0.98–2.96	0.059
Ependymal spread	1.16	0.57–2.40	0.680
Recurrence location (distant vs. local)	1.27	0.78–2.06	0.333
***Progression free survival***			
Age	1.01	1.00–1.03	0.155
Extent of resection (<90% vs. >90%)*	3.07	1.56–6.06	0.001
No initial therapy*	29.86	8.06–110.67	<0.001
Postoperative KPS (<80/>/=80)	1.21	0.80–1.82	0.374
Multifocal primary tumor	1.26	0.82–1.93	0.302
Primary tumor: contact to ventricle	0.92	0.58–1.46	0.716
Multifocal recurrence*	1.70	1.10–2.63	0.016
Recurrence: contact to ventricle	0.94	0.59–1.48	0.774
Ependymal spread	0.81	0.43–1.51	0.501
Recurrence location (distant vs. local)	0.87	0.58–1.31	0.510
***Post-progression survival***			
Age	1.01	0.99–1.03	0.461
No therapy for recurrent disease*	4.86	2.59–9.12	<0.001
KPS at recurrent disease (<80/>/=80)	1.48	0.90–2.42	0.123
Multifocal primary tumor	1.05	0.65–1.71	0.831
Primary tumor: contact to ventricle	0.96	0.56–1.65	0.874
Multifocal recurrence	1.42	0.84–2.41	0.193
Recurrence: contact to ventricle*	1.85	1.04–3.28	0.036
Ependymal spread*	2.97	1.41–6.26	0.004
Recurrence location (distant vs. local)*	1.75	1.10–2.79	0.019

CI: Confidence interval; *P < 0.05.

Subgroup analysis of patients with available MGMT-methylation status only revealed the following significant parameters: extent of resection < 90% (HR 3.22 [1.54–6.76], P = 0.002) and no therapy for recurrent disease (HR 4.21 [1.84–9.61], P = 0.001) and missing MGMT-methylation (HR 2.08 [1.07–4.03], P = 0.030. Multifocal tumor recurrence (HR 1.47 [0.75–2.86], P = 0.262) did not show statistical significance (Supplemental Table 1).

### Progression free survival

#### Univariate model

In univariate survival analysis extent of resection and initial adjuvant therapy were the strongest parameter for PFS (P < 0.001). Primary tumors with contact to ventricle showed significantly impaired progression free survival (P = 0.048). Interestingly, patterns of recurrent tumors did not show a correlation to PFS in this univariate model.

#### Multivariate model

The multivariate survival model included the following parameters: age, extent of resection, postoperative KPS (<80/>/ = 80), initial adjuvant therapy, multifocal growth and contact to ventricle of primary tumors, multifocal recurrence, contact to ventricle and ependymal spread at recurrence and location of recurrence. Apart from the known prognostic factors like extent of resection and therapy, also multifocal tumor recurrence was shown to be a prognostic factor for PFS (HR 1.70 [1.10-2.63], P = 0.016) (Table 4).

### Post-progression survival

#### Univariate model

**Figure 5:**
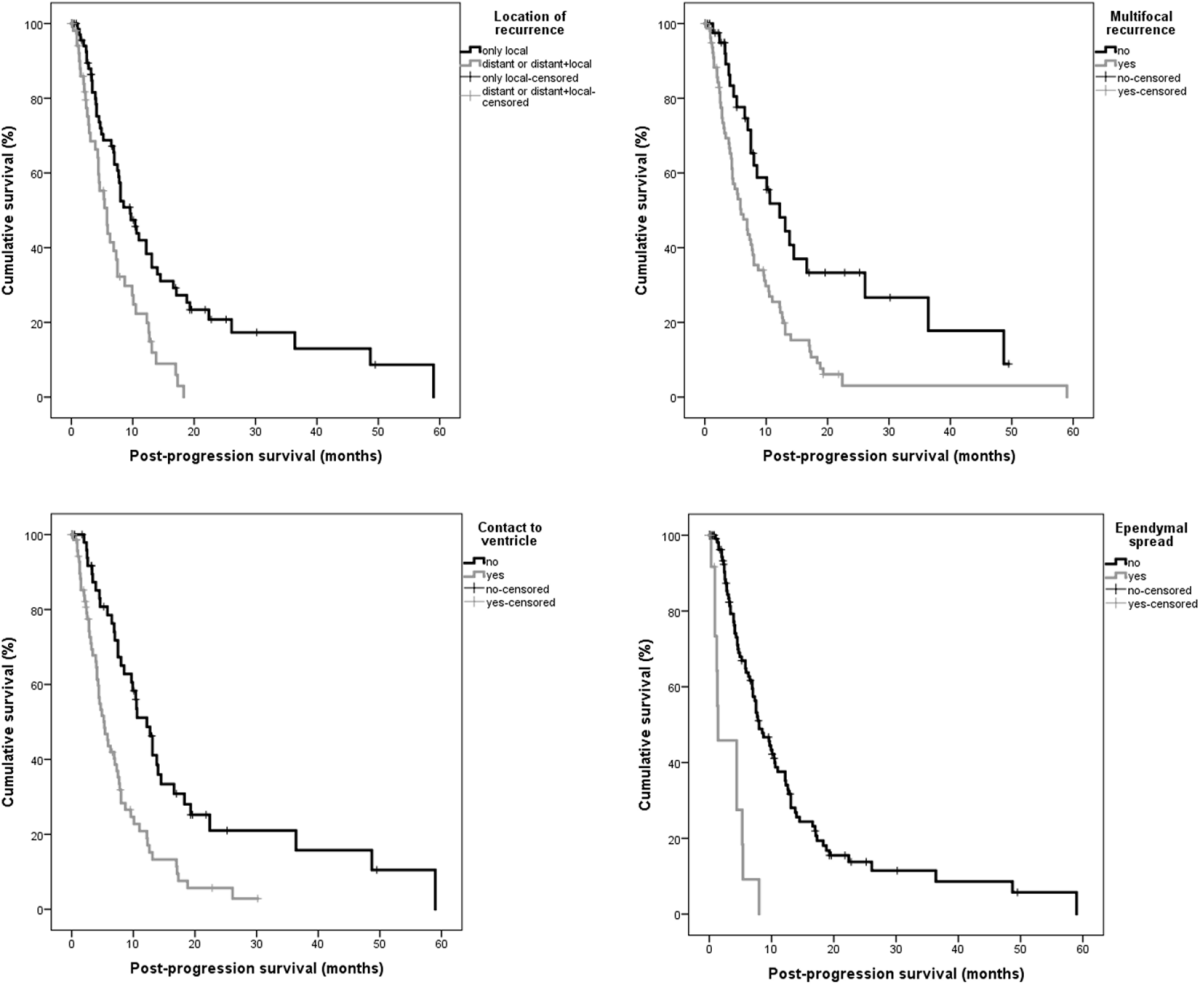
Post-progression survival estimates (Kaplan-Meier) for different recurrence patterns.

For post-progression survival the following recurrence patterns were significant prognostic factors: primary tumor with contact to ventricle (P = 0.048), location of tumor recurrence (P < 0.001), multifocal recurrence (P = 0.001), contact to ventricle (P < 0.001) and ependymal spread (P < 0.001) (Fig. 5).

Contact to dura (P = 0.582), homogenous/inhomogeneous (P = 0.686) and pattern of contrast enhancement (P = 0.384) of recurrent tumors were not shown as prognostic factors.

### Multivariate model

In the multivariate model contact to ventricle (HR 1.85 [1.04–3.28], P = 0.036), ependymal spread (HR 2.97 [1.41–6.26], P = 0.004) and location of recurrence (HR 1.75 [1.10–2.79], P = 0.019) remained as significant factors beneath the known variable therapy for recurrent disease (Table 4). Subgroup analysis of patients with available MGMT-methylation status showed similar results with contact to ventricle (HR 2.83 [1.35–5.94], P = 0.006), ependymal spread (HR 4.44 [1.77–11.11], P = 0.001) and recurrence location (HR 2.08 [1.22–3.55], P = 0.007) as significant factors (Supplemental Table 1).

## Discussion

This study demonstrates that postoperative infarct volume after initial glioblastoma surgery is associated with distinct recurrence patterns, and that these recurrence patterns themselves are significantly associated with overall and post-progression survival.

Patterns of tumor recurrence have been well studied in the last decade^13,14,21–23^. Most studies evaluated four recurrence types according to the classification of Pope *et al*.: local, distant, diffuse, multifocal^23^. The predominant type is local tumor recurrence^13,21,23–25^. Whereas local tumor recurrence is associated with a better prognosis, the remaining recurrence patterns are associated with impaired overall survival^15^. Most studies focused on recurrence patterns in correlation to specific therapy regimes, especially bevacizumab that was thought to be associated with a more invasive recurrence pattern and often FLAIR-only recurrence^22–24,26^.

In contrast to previous studies and the Pope criteria^13,23^ we analyzed only contrast-enhancing lesions, multifocal was defined as >/ = 2 contrast-enhancing lesions (in contrast to at least 3 lesions (contrast-enhancing or not) of the Pope classification) and are therefore not in accordance with these recurrence patterns. In addition to these previously defined criteria by Pope *et al*.^23^, we however also analyzed the patterns of contrast enhancement, the demarcation of the lesion and the homogeneity of the tumor, aiming to better characterize different recurrence patterns. However, in this study we could not observe significant differences between different patterns of contrast enhancement.

Our results are in agreement with previous studies showing that overall survival as well as post-progression survival is reduced in patients with a more aggressive recurrence pattern, including multifocal growth, contact to the ventricle, ependymal spread and distant tumor recurrence^12,15^. However, there is limited knowledge about the biology of these aggressive recurrence patterns. A recent study highlighted that postoperative infarct volume is independently of postoperative KPS associated with reduced overall survival^9^. This raises the questions which biological mechanisms underlie impaired survival in these patients with larger postoperative infarctions. The present study discloses an association between large postoperative infarct volumes and multifocal tumor growth, recurrence with contact to the ventricle and contact to the dura. The first two patterns were independently associated with impaired OS/PPS, which supports the hypothesis that postoperative ischemia triggers an aggressive growth pattern, leading to impaired survival. Although contact to the dura is not associated with impaired survival, this might also be a sign of tumor spread. Hypoxia is known to be a mediator for invasive tumor growth of glioblastoma^27–29^. Hypoxia-inducible factors (HIF), especially HIF1 and HIF2α are known to promote tumor growth and are therefore discussed as targets for therapy^30,31^. The results of our study confirm those of a previous study, which showed significantly more often diffuse/distant tumor recurrences in patients with postoperative infarctions^12^. These results suggest that reduction of postoperative infarct volume not only improves patients’ functional independence but also might reduce the risk of aggressive tumor growth and therefore impaired survival. One could assume that a more aggressive tumor resection might be the cause of larger infarct volumes and consequently diffuse tumor recurrence. However, in the present study extent of resection was not associated with aggressive tumor recurrence, suggesting that reduction of postoperative ischemia is of high importance^32^.

Another explanation might be that hypoxia has a negative effect on efficacy of radiotherapy. A recent study showed that initiating radiotherapy sooner than 24 days after surgery had a negative impact on overall survival and PFS, a finding that is also explainable by partial recovery of hypoxic areas after surgery^33^. Further studies that investigate the exact pathomechanisms that initiate this invasive, possibly hypoxia-mediated tumor growth will have to be performed to maybe reveal new therapy strategies.

Recent investigations also focused on the subventricular zone that contains neural stem cells initiating invasive tumor growth^16,17,34^. In this study we also observed significantly impaired survival for tumor growth at the ventricle and also a correlation between postoperative infarct volume and tumor recurrence with contact to the ventricle. Further studies might evaluate if there is also a correlation between these neural stem cells and hypoxia.

There are limitations of this study. The retrospective design is the main limitation of this study. This is also reflected by the different therapy regimes of the patients that were conducted at the discretion of the local expert committee and not in the framework of a study. Different therapy regimes and also the time frame between recurrence and therapy regimes might also affect radiological recurrence patterns. Especially bevacizumab is known to affect these patterns often showing a more diffuse tumor growth^22^. A recent study further did not show an association between recurrence patterns and survival in patients treated with bevacizumab^35^. However, in this study only 4/129 patients received bevacizumab before first tumor recurrence. The exact mechanisms and the influence of different therapy regimes on radiological recurrence patterns should be addressed in further studies.

Postoperative seizures are a common complication after glioma surgery. Post-ictal changes are known to show diffusion restriction on MR images. However, differentiation between post-ictal and ischemic changes are challenging, especially due to a high number of clinically hidden postoperative seizures.

Another bias might arise due to image analysis at different MRI scanners and therefore the use of different sequences.

Differentiation between tumor progression and pseudoprogression or contrast enhancement of postoperative ischemia are known to be a pitfall in radiological assessment of follow-up MRI^36^. Studies report that up to 20–30% of glioblastoma patients treated with radio- or chemotherapy present with pseudoprogression^37^. To address this bias in our study, the RANO criteria were used for radiological assessment, combined with advanced imaging methods (FET-PET, perfusion weighted imaging) and histopathological diagnosis of recurrence was obtained in almost half of the patient cohort.

Especially the measurement of small postoperative infarct volumes might introduce a bias, as differentiation between tumor recurrence and enhancing ischemia might be challenging. To account for this, we refer to the RANO criteria for tumor progression in this study^19^. Besides this, tumor recurrence was proven histopathologically in almost half of the patients and by advanced imaging methods in 23 cases.

## Conclusions

Postoperative infarct volume is associated with recurrence patterns (multifocal, contact to ventricle/dura) which might be an explanation for the impaired overall survival of patients with large postoperative infarctions. A possible pathomechanism might be hypoxia-induced tumor growth. Further studies investigating this relationship might have to be performed to find therapy regimes for this aggressive tumor type with poor prognosis.

## Electronic supplementary material

Supplemental Table 1
