# Supplementary material for: Retrospective Analysis of Radiological Recurrence Patterns in Glioblastoma, Their Prognostic Value And Association to Postoperative Infarct Volume
Source: Sci Rep. 2018 Mar 14;8:4561. doi: 10.1038/s41598-018-22697-9 (PMC5852150; doi:10.1038/s41598-018-22697-9)
Supplement: Supplementary file 1 — Supplemental Table 1 [file 41598_2018_22697_MOESM1_ESM.pdf]

Retrospective Analysis of Radiological Recurrence Patterns in Glioblastoma, Their Prognostic Value And Association to Postoperative Infarct Volume

Stefanie Bette (MD), Melanie Barz (MD), Thomas Huber (MD), Christoph Straube (MD), Friederike Schmidt-Graf (MD), Stephanie E. Combs (MD), Claire Delbridge (MD), Julia Gerhardt (MD), Claus Zimmer (MD), Bernhard Meyer (MD), Jan S. Kirschke (MD), Tobias Boeckh-Behrens (MD), Benedikt Wiestler (MD), Jens Gempt (MD)

Supplemental Table 1 Multivariate survival analysis for the subgroup of patients with available MGMT-methylation status (n=95)

Parameter	Hazard Ratio	95% CI	*P* value
Overall survival			
Age	1.01	0.99-1.03	0.409
Extent of resection (<90% vs. >90%)*	3.22	1.54-6.76	0.002
No therapy for recurrent disease*	4.21	1.84-9.61	0.001
KPS at recurrent disease (<80/>/=80)	0.95	0.53-1.70	0.854
MGMT-unmethylated*	2.08	1.07-4.03	0.030
Multifocal primary tumor	0.89	0.49-1.65	0.721
Primary tumor: contact to ventricle	1.39	0.70-2.73	0.345
Multifocal recurrence	1.47	0.75-2.86	0.262
Recurrence: contact to ventricle	1.94	0.89-4.24	0.096
Ependymal spread	2.00	0.81-4.97	0.134
Recurrence location (distant vs. local)	1.13	0.66-1.94	0.652
Progression free survival			
Age	1.02	1.00-1.04	0.073
Extent of resection (<90% vs. >90%)*	2.65	1.24-5.69	0.012
No initial therapy*	71.88	10.17-508.09	<0.001
Postoperative KPS (<80/>/=80)	1.29	0.78-2.14	0.326
MGMT-unmethylated	2.20	1.28-3.78	0.004
Multifocal primary tumor	0.76	0.45-1.30	0.324
Primary tumor: contact to ventricle	1.48	0.85-2.56	0.163
Multifocal recurrence	1.53	0.91-2.59	0.112
Recurrence: contact to ventricle	0.71	0.38-1.35	0.296
Ependymal spread	1.49	0.70-3.19	0.305
Recurrence location (distant vs. local)*	0.59	0.36-0.95	0.028
Post-progression survival			
Age	1.01	0.99-1.03	0.292
No therapy for recurrent disease*	3.26	1.48-7.19	0.003
KPS at recurrent disease (<80/>/=80)	1.47	0.82-2.63	0.196
MGMT-unmethylated	1.75	0.90-3.42	0.102
Multifocal primary tumor	0.97	0.53-1.79	0.922
Primary tumor: contact to ventricle	0.66	0.35-1.25	0.206
Multifocal recurrence	1.04	0.54-2.01	0.912
Recurrence: contact to ventricle*	2.83	1.35-5.94	0.006
Ependymal spread*	4.44	1.77-11.11	0.001
Recurrence location (distant vs. local)*	2.08	1.22-3.55	0.007

CI: Confidence interval; * *P*<.05
